# Conversational AI for Child Abuse Detection Through Multistage Counseling: Model Development and Validation Study

**DOI:** 10.2196/86536

**Published:** 2026-07-10

**Authors:** Hyun-Young Moon, Youn-Gyu Jin, YoonJu Kim, Gwang-Cheol Lee, Hyeontaek Oh, Hyun A Kim, Dinara Aliyeva, Hyunjoo Na, Kang-Min Kim

**Affiliations:** 1 Department of Artificial Intelligence, The Catholic University of Korea, Bucheon, Republic of South Korea; 2 Department of Psychology, The Catholic University of Korea, Bucheon, Republic of South Korea; 3 Department of Computer Science, College of Arts & Sciences, University of North Carolina at Chapel Hill, Chapel Hill, NC, United States; 4 College of Nursing, The Catholic University of Korea, Seoul, Republic of South Korea; 5 Department of Software Convergence, Kyung Hee University, 1732 Deogyeong-daero, Giheung-gu, Yongin, Gyeonggi, 17104, Republic of South Korea, 82 10-3253-8767

**Keywords:** child abuse detection, conversational artificial intelligence, counseling, large language model, pretrained language model

## Abstract

**Background:**

Child abuse severely disrupts the healthy growth and development of children, resulting in long-term physical as well as emotional consequences. In real-world settings, despite the continuous increase in reported abuse cases, the chronic shortage of certified child abuse professionals has significantly increased the workload of individual counselors, making timely intervention increasingly difficult.

**Objective:**

This study aims to reduce the workload of counselors in real-world counseling settings by supporting counseling and child abuse detection processes. We propose a conversational artificial intelligence–based framework, Conversational Artificial Intelligence for Child Abuse Detection (CACAD), which conducts counseling with children and detects child abuse during the counseling process.

**Methods:**

CACAD uses a large language model (LLM) to conduct counseling and to detect 4 types of child abuse: neglect, emotional, physical, and sexual. During the question generation process, the LLM serves as the primary agent, supported by 2 auxiliary modules. In the process of counseling, the LLM first determines whether a child’s response provides sufficient information to understand the current situation. If the response is deemed insufficient, the LLM generates follow-up questions to elicit additional information while preserving the context of the previous question. Once sufficient information is obtained, the next question category prediction module predicts the most appropriate category for the subsequent question and passes it to the LLM, enabling more flexible guidance of the counseling flow. In parallel, the abusive question detection module filters out potentially harmful or inappropriate questions to protect children. For abuse detection, CACAD uses an instruction-tuned LLM specialized for child abuse detection, and uncertainty quantification is applied to dynamically flag cases as pending review for counselor confirmation.

**Results:**

Experimental results using a Korean child-and-adolescent counseling dataset show that CACAD achieves strong performance in child abuse detection, with an exact match of 0.907 and a macro*–F*_1_-score of 0.939. In addition, CACAD demonstrates effective performance in counseling-related tasks, including next question category prediction and abusive question detection, contributing to coherent and safe counseling interactions. Human evaluation by domain experts further confirms the reliability of CACAD in counseling sessions, and uncertainty-based selective prediction allows the system to dynamically identify cases requiring human review.

**Conclusions:**

These findings demonstrate that LLM-based conversational agents can reliably perform child abuse detection within counseling conversations in real-world settings. The results further indicate that such systems can support counseling processes by integrating abuse detection, safe question generation, and uncertainty-aware decision handling in a unified framework.

## Introduction

### Background

Child abuse refers to acts by adults (eg, parents and caregivers) that involve violence or maltreatment, which can harm a child’s health or well-being, or otherwise impede normal development. According to a joint report published in 2020 by the United Nations [1], the World Health Organization [2], and the United Nations Children’s Fund [3], approximately 1 billion children worldwide are estimated to have experienced various forms of violence, including neglect, emotional, physical, or sexual abuse [4]. Child abuse is an increasingly widespread global issue, with various forms of abuse leading to long-term physical and emotional consequences [5-7]. Therefore, early identification and intervention remain critical in mitigating these harms [8].

Despite growing international awareness of child abuse, many countries continue to struggle with the implementation of effective prevention and response systems [9]. To address this issue, various methods [10,11] have been attempted to support the early identification and intervention of child abuse. However, these methods commonly rely heavily on the direct involvement of professionals, and the repetitive and intensive work structure in case management and counseling inevitably leads to staff burnout. According to the study by Fuseini [12], child protection social workers worldwide are facing severe burnout, characterized by excessive workloads, emotional exhaustion, and professional disillusionment. This situation contributes to high turnover rates, undermining the effectiveness of child abuse response systems, while chronic staff shortages further exacerbate the workload of the remaining social workers [13].

**Figure 1.**
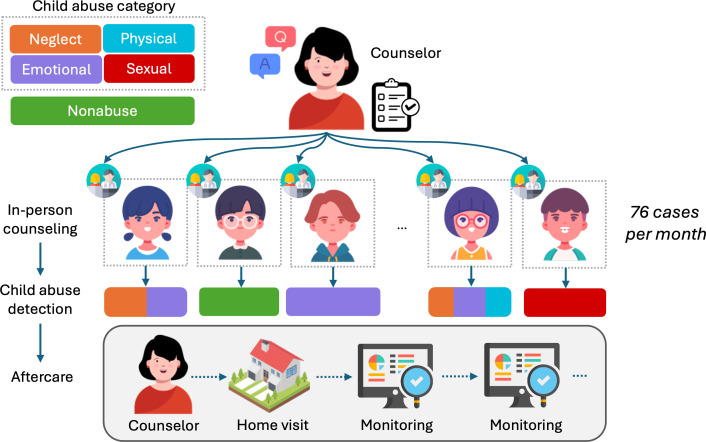
Illustration of the excessive workload faced by an individual child counselor. A counselor in South Korea manages an average of 76 cases per month. Each case involves a demanding range of responsibilities, including in-person counseling and abuse detection, as well as intensive aftercare such as home visits and continuous monitoring.

This global challenge is mirrored in South Korea. Despite ongoing efforts to protect abused children, the number of suspected child abuse reports nearly tripled from 15,025 in 2014 to 45,771 in 2023, and the shortage of counseling personnel remains unresolved [14]. Child counselors in the United States handled an average of 15 cases per month in 2021, whereas their South Korean counterparts managed 76 cases in 2020 [15]. Such an excessive caseload per counselor poses a significant challenge in both assessing child abuse and implementing complex aftercare interventions, as illustrated in Figure 1. The resulting strain may compromise the quality of care and increase the risk of overlooking critical signs of abuse. This highlights the growing need for effective tools that can conduct child abuse counseling, detect signs of abuse, and triage cases, thereby alleviating the burden on counselors.

Recent advancements in large language models (LLMs) have shown that LLMs demonstrate human-level capabilities in natural language understanding and generation [16-19], revealing the potential of conversational artificial intelligence (AI) systems. In particular, conversational AI systems such as ChatGPT (OpenAI) [20], DeepSeek (DeepSeek) [21], and Gemini (Google DeepMind) [22] extend beyond basic question-answering, demonstrating the ability to generate contextually appropriate responses and accurately interpret emotional cues within sophisticated counseling. For instance, LLM-based counseling systems match the performance of human counselors in alcohol addiction interventions [23], excelling in linguistic appropriateness and response safety. In addition, significant improvements in empathy, emotional regulation, and conversation quality have been observed in mental health screening and cognitive behavioral therapy contexts [24,25].

Nevertheless, the application of conversational AI in the field of child abuse counseling remains largely unexplored. The existing approach has primarily relied on rule-based counseling systems in which experts predefine the questions and their sequence. While this system ensures procedural consistency, it lacks flexibility, as it continues to follow a predetermined flow of questions regardless of the child’s responses. Moreover, the current system detects only a single type of abuse [26]. Prior studies [27,28] have shown that children frequently experience multiple forms of abuse, underscoring the need for a framework capable of multilabel prediction. However, applying LLMs to counseling introduces additional risk. LLMs can generate inaccurate predictions and, more critically, produce inappropriate or abusive questions during counseling [29,30]. These risks can potentially cause secondary harm to children and thus represent a critical limitation in applying AI to high-stakes counseling domains such as child abuse counseling and abuse detection.

### Goal of This Study

To address this challenge, we propose a novel framework, Conversational Artificial Intelligence for Child Abuse Detection (CACAD), which consists of 2 stages. In the first stage, the LLM serves as the primary agent of counseling, generating the next question while being supported by 2 auxiliary modules to ensure flexibility and safety. In the second stage, CACAD analyzes the entire conversation and performs multilabel classification (MLC) to determine the presence of each abuse category. To reduce misclassification, CACAD quantifies prediction uncertainty, and cases that exceed a predefined threshold are flagged for human counselor review.

In this study, we evaluate the effectiveness of CACAD through counseling sessions and abuse detection experiments. We use the child and adolescent counseling dataset [31] provided by AI-Hub [32], a public AI training data platform operated by the National Information Society Agency of Korea [33].

In summary, our contributions are 3-fold:

1. We propose CACAD, a framework that alleviates the burden on human counselors through automated counseling and the detection of child abuse indicators.
2. We develop a 2-stage framework that integrates 2 auxiliary modules to guide LLM-based question generation and performs MLC with uncertainty quantification to support reliable abuse detection.
3. We demonstrate the effectiveness of CACAD through human evaluation and quantitative experiments, showing its superior counseling quality and child abuse detection performance, which outperform all baselines on a real-world child and adolescent counseling dataset.

### Prior Work

#### Conversational AI in Counseling

Early counseling chatbots operated in a rule-based manner, classifying user utterances by intent or emotion and selecting responses from a predefined set [34,35], or by classifying emotional states and providing them to counselors as decision support [36,37]. However, these studies commonly had the limitation of not being able to generate responses directly. This limitation was alleviated with the advent of LLMs, which can understand context and generate new responses. Recently, LLM-based approaches have been widely adopted, with studies in mental health counseling reporting improvements in accessibility through empathetic responses and continuous support [38,39], while career counseling has shown comparable effectiveness to traditional approaches in supporting decision-making and providing personalized advice [40]. Research focusing on children has also emerged, primarily targeting psychoeducation and coping skill training [41,42]. Nevertheless, few studies have applied conversational AI to counseling for child abuse victims, leaving a critical research gap that this study aims to address.

#### Child Abuse Detection

Previous research on child abuse detection has explored 3 primary approaches involving image analysis, unstructured text processing, and structured data modeling [43]. Representative image-based studies have involved training convolutional neural networks on X-ray images to classify physical abuse [44] and examining self-portraits to identify signs of sexual abuse [45]. In the domain of unstructured text, researchers have applied natural language processing techniques to clinical notes, radiology reports, and emergency room records from electronic medical records to uncover early indicators of physical abuse [46-48]. Structured data approaches combined demographic attributes, clinical information, and child protection records to construct predictive models such as artificial neural networks [47,49]. However, most existing studies focus either on a limited range of child abuse categories, such as physical or sexual abuse, or only determine whether child abuse has occurred. In this study, we propose a model that analyzes counseling data collected from real sessions and detects 4 categories of child abuse through MLC while incorporating uncertainty quantification to enhance the reliability of its prediction. The model aims to detect specific patterns of child abuse embedded in natural counseling.

## Methods

### Ethical Considerations

The data used in this study consist of counseling records publicly released on AI-Hub, an open data platform operated by the Ministry of Science and ICT of the Republic of Korea. We use this dataset for research purposes in strict accordance with the AI-Hub open data usage guidelines [50]. During the data construction process, counselors removed all personally identifiable information related to the children and their guardians as part of the preprocessing procedure. All counseling materials generated during the sessions were recorded and stored without any personal identifiers, and each counseling case was distinguished solely by a randomly assigned identifier created for dataset construction purposes. As a result, throughout the entire data collection and release process, no information enabling the identification of individual children was retained. At the time of public release, the dataset had already undergone a comprehensive deidentification process, ensuring that all personal information was irreversibly removed in advance. To ensure the ethical appropriateness of our data usage, we directly contacted the data management office at AI-Hub for verification. We were informed that secondary analysis of publicly available deidentified data does not require institutional review board review or formal ethical approval. Furthermore, the human evaluation, conducted through professional role-playing, was performed without the direct participation of children or interactions with human subjects. The data management authority also confirmed that this evaluation method does not require ethical review or institutional review board approval.

### Dataset Construction

To support both the counseling and child abuse detection stages of the CACAD framework, we construct 3 task-specific datasets based on real-world counseling records of children and adolescents. The raw data consist of 3596 structured question-answer counseling records between counselors and children (1797 male and 1799 female), including quantitative scores and counselor comments. Each record corresponds to a single counseling session associated with a unique child identifier. After excluding cases with missing data, the final dataset consists of 3205 records. To prevent data leakage and ensure independence between splits, the dataset was divided into training, validation, and test sets at the child level following an 8:1:1 ratio using stratified sampling. Table 1 presents the label distribution of the child abuse detection dataset across the 4 categories.

**Table 1.** Child abuse detection dataset distribution by label and category^a^.

Category and label	Training	Validation	Test
Neglect			
Nonabuse	2160	265	280
Abuse	404	55	41
Emotional			
Nonabuse	1829	239	225
Abuse	735	81	96
Physical			
Nonabuse	2061	267	253
Abuse	503	53	68
Sexual			
Nonabuse	2343	286	288
Abuse	221	34	33

a The sets were partitioned (8:1:1) using stratified sampling based on multilabel combinations to preserve the underlying label distribution and mitigate bias toward specific combinations.

### Next Question Category Prediction Dataset

To ensure effective child abuse detection, LLMs must explore diverse topics to identify subtle indicators of abuse. However, without context-appropriate guidance, an LLM might generate repetitive, topic-confined, or overly simple follow-ups based on the child’s prior responses. Such limitations are misaligned with the objectives of child abuse detection, which require probing diverse topics to uncover subtle cues. To address this limitation, we constructed the next question category prediction (NQCP) dataset to facilitate context-driven question generation. For each abuse type, we extract and embed counselor questions from the training split using the Korean sentence-transformer model jhgan/ko-sroberta-multitask [51]. We then apply hierarchical density-based spatial clustering of applications with noise [52] to generate question categories. However, hierarchical density-based spatial clustering of applications with noise often produces an excessive number of fine-grained clusters, separating semantically similar questions due to lexical or phrasing variations. To mitigate this, clusters are merged when the cosine similarity between centroids exceeds a predefined threshold. Questions from the validation and test splits are assigned to the most similar category by comparing their embeddings to the cluster centroids. In addition, questions initially labeled as noise are reassigned to the nearest cluster if their similarity to the centroid surpasses a predefined threshold; otherwise, they are excluded to maintain label quality. To ensure proper conversation termination, a special token |end| is appended to the final counselor question in each conversation and assigned to a dedicated category. This process yields 13 categories for neglect, 10 for emotional abuse, 11 for physical abuse, and 7 for sexual abuse. The resulting clusters are mapped back to the counseling records to serve as ground truth labels. We then construct the NQCP dataset by extracting training samples at each counselor’s turn within the counseling conversations. For each sample, the input is defined as the accumulated conversation history up to a given child response (eg, Q1-A1 or Q1-A1-Q2-A2), while the prediction target is the cluster category of the subsequent counselor question (eg, Q2 or Q3). When the input conversation history exceeded 512 tokens, it was truncated by removing the oldest utterances first to preserve the most recent conversational context. This refinement process results in a final dataset consisting of 41,938 samples.

### Abusive Question Detection Dataset

To address the possibility that CACAD may generate abusive questions that could cause secondary harm, blame the child, or justify abuse, we construct an abusive question detection dataset. First, we select one representative question from each category based on the question categories constructed in the NQCP dataset. These representative questions serve as reference inputs for GPT-4o [53] to generate synthetic abusive questions. We generate synthetic data based on 5 types of abusive questions defined by 2 domain experts. The defined types of abusive questions are as follows:

1. Suggestive type: questions that guide the child by offering predetermined answers.
2. Self-blaming type: questions that induce inappropriate feelings of guilt.
3. Authority-imposing type: questions expressed in a commanding or directive tone.
4. Emotion-ignoring type: questions that overlook or dismiss the emotions of the child.
5. Adult-centric type: questions that interpret situations solely from the perspective of adults

For each type-specific prompt, we explicitly instruct the model to transform original questions to reflect the corresponding abusive characteristics. Using each prompt, we generate 25 questions per abuse category, yielding 100 questions per prompt and a total of 500 abusive questions. These generated questions are thoroughly reviewed by 2 domain experts through a joint consensus-based inspection process, during which their expressions and contexts are refined to ensure that they are recognized as abusive in actual counseling environments. Finally, we extract 500 nonabusive questions from the original dataset, 125 questions for each abuse category, and incorporate them into the final dataset, resulting in a complete set of 1000 questions.

### Child Abuse Detection Dataset

To determine the presence of child abuse based on full counseling sessions, we construct a multilabel binary classification dataset. For each counseling case, independent binary labels for 4 types of abuse are assigned based on scores and comments provided directly by counselors in the original dataset. The labeling criteria are established through expert review of these scores and comments. The scores and corresponding comments are presented in Table 2.

1. Neglect: 0‐3 → nonabuse, 4‐10 → abuse
2. Emotional abuse: 0‐4 → nonabuse, 5‐10 → abuse
3. Physical abuse: 0‐4 → nonabuse, 5‐10 → abuse
4. Sexual abuse: 0‐4 → non-abuse, 5‐10 → abuse

**Table 2.** Scoring criteria and clinician comments for child abuse severity across 4 categories in the original Korean child and adolescent counseling dataset construction provided by AI-Hub^a^.

Category	Indicators (score)
Neglect	Receives appropriate care (0 points) Lacks care, leading to difficulty in meeting physical needs (6 points) Lacks care, leading to difficulty in meeting emotional needs (6 points) Lacks care, leading to difficulty in meeting both physical and emotional needs (8 points) Other: [Under 25 characters] Example: Other: Although the child experienced abuse in the past, they are currently receiving care and protection in a safe facility (3 points) Other: Neglect regarding meals due to disciplinary action (4 points)
Emotional	No signs of emotional abuse are inferred (0 points) Suspected emotional abuse possibly leading to depression or psychological discomfort (5 points) Suspected emotional abuse possibly leading to severe distress or psychotic symptoms (10 points) Other: [Under 25 characters] Example: Other: Although the child experienced abuse in the past, they are currently receiving care and protection (4 points) Other: The child has experienced emotional abuse until recently (5 points)
Physical	No signs of physical abuse are inferred (0 points) Physical punishment perceived as related to discipline (5 points) Excessive punishment or suspected abuse that requires careful observation (8 points) Severe physical punishment or abuse that requires active intervention (10 points) Other: [Under 25 characters] Example: Other: Although the child experienced abuse in the past, they are currently receiving care and protection in a safe facility (3 points) Other: The child experienced violence from their father but is currently in a stable condition (4 points) Other: It is believed that excessive punishment or abuse occurred in relation to discipline (6 points)
Sexual	No signs of sexual abuse are inferred (0 points) Experienced situations or actions possibly involving sexual abuse (5 points) Sexual abuse is strongly suspected, and emotional support is needed (8 points) Severe sexual abuse requiring urgent intervention, support, and legal action (10 points) Other: [Under 25 characters] Example: Other: Sexual abuse is suspected, but the exact circumstances are unclear (6 points) Other: There are signs suggesting possible sexual abuse, requiring careful observation and support (6 points)

a The table summarizes the original severity indicators and scoring scheme, with clinician comments selected from cases near the abuse and nonabuse threshold to explain the rationale for the abuse and nonabuse distinction based on category-specific score cutoffs.

### Our Framework

#### Overview

**Figure 2.**
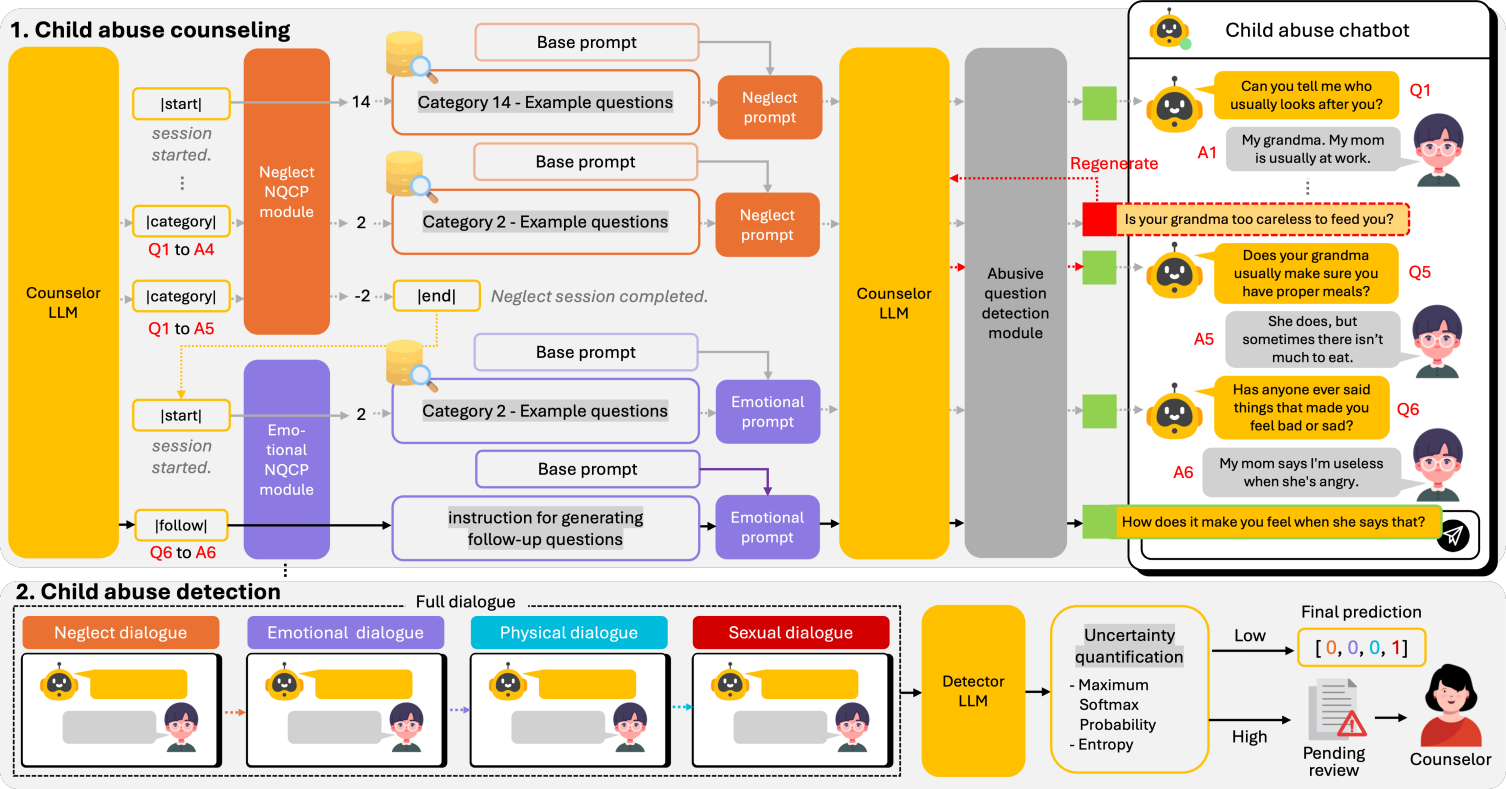
Overview of the Conversational Artificial Intelligence for Child Abuse Detection framework. The framework consists of two stages. (1) Child abuse counseling stage: a 2-step generation process driven by a counselor’s large language model. (A) The conversation flow is controlled by |category| and |follow| token, transitioning to a new topic via the next question category prediction module when information is sufficient or generating follow-up questions. (B) An abusive question detection module evaluates the generated questions to ensure safety by filtering offensive content and triggers regeneration. (2) Child abuse detection stage: a multilabel abuse detection based on full counseling sessions using uncertainty quantification to flag ambiguous cases as “pending” for expert review, thereby enhancing diagnostic reliability. LLM: large language model.

Our framework, CACAD, consists of 2 sequential stages, as illustrated in Figure 2. The first stage, enhancing counseling through question flow control and safety filtering, focuses on controlling question flow and ensuring safe interactions. In this stage, the NQCP module determines the category of the next question to generate contextually appropriate prompts, dynamically alternates between category prediction and follow-up questioning to maintain a natural conversational flow, and filters out abusive questions to ensure safe interactions. The second stage, child abuse detection via instruction tuning and uncertainty quantification, focuses on detecting multiple abuse types, applying uncertainty thresholds to reduce overconfident errors, and enabling expert intervention in ambiguous cases.

#### Enhancing Counseling Through Question Flow Control and Safety Filtering

##### NQCP

To build the NQCP module, we fine-tune a pretrained language model (PLM) using the previously constructed dataset of counselor questions. Through this training process, the next question category classification model learns how counseling context influences transitions between categories, thereby enabling coherent and contextually appropriate counseling flow. We train a separate PLM for each abuse type (neglect, physical, emotional, and sexual), allowing each classification model to specialize in the distinct progression patterns characteristic of its category. The output of each classification model provides the next question category, which is subsequently integrated into the LLM prompt during counseling.

##### Dynamic Counseling Between Category Prediction and Follow-Up Questioning

Effective counseling with children requires a question flow that flexibly adapts to the child’s responses and naturally guides the counseling process. To achieve this, the LLM outputs either a |category| or |follow| token based on the prior counseling context. As illustrated in Figure 2, when the LLM outputs a |category| token, the CACAD invokes the NQCP prediction module, which determines the next question category and provides representative example questions. These examples are inserted into the prompt, guiding the LLM to generate contextually appropriate next questions. In contrast, when the LLM outputs a |follow| token, the CACAD generates additional follow-up questions within the same category. This occurs not only when the child’s response is insufficient or ambiguous, but also when further elaboration is needed to capture specific details. For instance, in Figure 2 (Q6-A6), although the child reveals a sign of emotional abuse, the CACAD issues a |follow| token to elicit additional context such as frequency, severity, or specific circumstances. However, as repeated follow-up interactions can substantially increase the length of the conversation history, during counseling, the NQCP module predicts the next question category using only question-answer pairs that exclude interactions generated by |follow| tokens. If the input still exceeds 512 tokens, the oldest utterances are removed first. In addition, when the NQCP module predicts a category that has already been selected in a previous step, the system instead chooses the next most probable category to ensure progressive coverage of distinct counseling stages. At this stage, each prompt used for question generation is followed by the entire conversation history, enabling the LLM to generate the next question that is contextually appropriate. Each counseling session proceeds sequentially through 4 abuse types, enabling the LLM to consistently track the counseling flow and maintain appropriate questioning strategies throughout the session. Details of the prompts are provided in Table 3.

**Table 3.** Prompt templates used in the proposed framework, including prompts for abuse detection, category or follow-up verification, the base prompt provided to the baselines and category question generation, category question generation, and follow-up question generation^a^.

Method	Prompt
Abuse detection instruction tuning prompt	You are a child abuse assessment counselor, and your task is to determine whether actual abuse has occurred based on the conversations for each category. The following is a counseling conversation with a child. The conversation is divided into 4 categories (neglect, emotional abuse, physical abuse, and sexual abuse). Each category begins with a tag in the form <|category name|>, followed by a Q&A-style conversation related to that category. After <|pred|>, a label indicates whether abuse is present for each category in the following format: [neglect, emotional abuse, physical abuse, sexual abuse] Use 1 if the case corresponds to abuse and 0 if it does not. ## Example {examples} Now, review the given conversation and determine whether abuse has occurred. {conversation}
Category or follow token prediction prompt	You are a professional counselor providing child abuse counseling to children and adolescents regarding {abuse_type}. Your role is to determine whether the child’s response provides sufficient information to the previous question or whether additional follow-up questions are needed. ##Decision criteria # |category| (sufficient information) Select this only if all of the following conditions are met: - The response directly answers the question - It includes at least one concrete piece of information about the situation, behavior, or experience - The response clearly fulfills the purpose of the question - You can move on to the next step without asking additional questions # |follow| (additional question needed) Select this if any one of the following applies: - The response contains no information or is overly brief (eg, “I don’t know,” “No,” “Yes”) - The response is not directly related to the question - The response suggests the question was not understood - The response is ambiguous or difficult to judge ##Output choice - |category| - |follow| **The output must be exactly one of the output choices shown above.** Do not output any other words, sentences, symbols, spaces, or line breaks. Do not output explanations, reasons, commentary, or any additional text.
Base prompt	You are a professional counselor providing child abuse counseling to children and adolescents regarding {abuse_type}. ## conversation rules -Use a friendly and warm tone. -Ask exactly one question in each turn. -Use simple language and short sentences that children and adolescents can understand. -Ask questions indirectly and naturally, so the child feels comfortable. -Do not deny or doubt the child’s responses; acknowledge them and continue the conversation -Maintain the flow of the previous conversation and connect naturally
Baseline prompt	{Base prompt} ##Counseling goals - Collect specific factual information about the {abuse_type} situation - Assess the child’s current safety status - Understand the child’s emotions and difficulties ##Conversation Termination If you output the |end| token, the conversation will terminate immediately. When you determine that the situation does not involve abuse or that sufficient information has been obtained, output the |end| token **without any additional text**. The conversation should be wrapped up naturally within a maximum of 10 turns, and on the 11th turn, it must output only |end| ##{abuse_type} conversation examples {example_conversations} Now generate the question by referring to these conversation rules and flow.
Category question prompt (CACAD^b^)	{Base prompt} ##Counseling goals - Collect specific factual information about the {abuse_type} situation - Assess the child’s current safety status - Understand the child’s emotions and difficulties ## Category information {category_definition} {category_example_questions} - The meaning, intent, and information scope of the question must never be changed. -The wording may vary, but the core information being asked must be 100% identical to that of the reference question. ##{abuse_type} conversation examples {example_conversations} Now generate the question by referring to these conversation rules and flow.
Follow-up question prompt (CACAD)	{Base prompt} The child’s response is not sufficient at this point, and the information intended to be obtained from the previous question cannot yet be clearly identified. Your role is to progressively supplement only the missing information while maintaining the original intent of the previous question. ## follow-up question generation order Follow-up questions must strictly follow the order below. Stage 1: Experience clarification If the experience is not yet specific enough, first clarify what actually happened. possible question types: -When it happened -Where it happened -What happened At this stage, do not ask about responses, help, or resolution. Stage 2: Response and support check Only after the experience has been clearly identified, check whether any help or protection was provided in that situation. possible question types: Whether there was an adult who helped at the time Whether there was someone the child could contact Whether the child had to handle it alone Generating Stage 2 questions before the experience is clearly established is not allowed ##{abuse_type} conversation examples {example_conversations} Now generate the question by referring to these conversation rules and flow.

a The prompts were originally developed in Korean and are presented here as English translations.

b CACAD: Conversational Artificial Intelligence for Child Abuse Detection.

##### Abusive Question Detection

To build the abusive question detection module, we fine-tune a PLM using the previously constructed abusive question detection dataset. Through this process, the abusive question classification model learns to determine whether questions generated by the LLM contain offensive or inappropriate expressions. As illustrated by the red boxes in Figure 2, the classification model reviews each generated question before it is delivered to the child. If inappropriate or harmful expressions are detected, the detection module requests the LLM to regenerate the question. This process is repeated until a safe and contextually appropriate question is obtained, ensuring that only safe outputs are used in sensitive counseling scenarios. While this iterative approach maximizes system safety, it may introduce latency or risk of infinite loops in real-time counseling. To mitigate these operational risks while maintaining system stability, we use a fallback mechanism that is triggered when regeneration for the same question exceeds 2 attempts. If the predicted token corresponds to a category and question regeneration exceeds 2 attempts, the system directly selects the most appropriate question from the corresponding category without modification. Conversely, if the predicted token corresponds to a follow token, the system halts further regeneration and proceeds to the next question category. By incorporating this filtering step, the CACAD prevents the generation of harmful or abusive prompts that could otherwise cause secondary harm to children.

### Child Abuse Detection via Instruction Tuning and Uncertainty Quantification

Once the counseling stage is completed, CACAD proceeds to the second stage in which CACAD detects the presence of child abuse based on the counseling content. To this end, we perform instruction tuning [54] on an LLM using the child abuse detection dataset. The prompt format used for instruction tuning is provided in Table 3. The abuse detection task follows an MLC approach, where the model independently predicts either nonabuse or abuse for each of the 4 abuse categories. In addition, to reduce misclassification, we incorporate uncertainty quantification techniques into the inference process. We compare 2 methods: maximum softmax probability (MSP) [55] and entropy [56]. Under MSP, lower softmax probabilities indicate greater uncertainty, whereas in entropy, higher values directly reflect greater uncertainty. Counselors can choose between these 2 methods depending on their preferences. If a prediction exceeds the predefined uncertainty threshold for either method, the entire counseling session is flagged as pending review and forwarded to a human counselor for further evaluation. This design accounts for the possibility of multiple types of abuse occurring simultaneously and supports careful expert intervention when necessary. It also mitigates errors caused by the generative instability of LLMs, thereby improving the overall reliability and safety of the framework.

### Evaluation Metrics

#### Child Abuse Counseling Evaluation

To evaluate the quality of counseling conducted by CACAD, we perform a human evaluation using a Gradio web interface. Three domain experts participate in the evaluation. We provide each expert with separate access links to 4 different systems and do not disclose the identity of each system in advance. Every link follows a standard conversation protocol where the systems conduct multiturn dialogues that cycle through all 4 abuse types in a predefined order. The evaluators freely choose the order in which they access the links and independently rate each conversation on a 7-point scale according to the 3 evaluation criteria defined in Table 4 (diversity, informativeness, and similarity), without using any consensus procedure or majority voting. Each expert assumes the role of a child or adolescent and conducts at least 5 counseling sessions per system, including CACAD and the baseline comparison models. A “turn” consisted of one assistant question and one child response. While the baseline terminated most dialogues at 10 turns via prompt instructions, CACAD dynamically terminated conversations within 6‐15 turns based on the interaction and the NQCP module. After completing multiple sessions across different models, the experts independently evaluated the overall quality of the responses generated by each system from a clinical perspective. To assess the agreement among the 3 evaluators, interrater reliability was measured using the intraclass correlation coefficient (ICC) [57]. The detailed definitions of each criterion are as follows:

1. Question diversity: evaluates whether the conversation avoids semantically redundant questions and spans a broad range of topics.
2. Informativeness: evaluates whether follow-up questions, grounded in preceding user responses, effectively elicit substantive information.
3. Conversational similarity with real counseling: evaluates whether the generated conversation mirrors authentic counseling discourse in linguistic expression, informational organization, and conversational flow.

**Table 4.** Human evaluation criteria for the quality of CACAD^a^^,^^b^.

Criterion	Scoring guidelines
Diversity	Questions are highly repetitive in topic and expression, making the conversation monotonous (1). Slight variation in wording, but the questions are semantically repetitive and limited in scope (2). Minor variation in phrasing exists, but the conversation remains focused on a single narrow topic (3). Occasional topic changes appear, but the questions still share similar structures or intent (4). Different topics are explored to some extent, though noticeable redundancy in expression remains (5). Most questions are distinct in both topic and expression, with minimal repetition and coverage of diverse areas (6). All questions are clearly distinct in meaning, structure, and focus, with smooth and natural topic transitions (7).
Informativeness	Questions are irrelevant to abuse detection and disrupt the conversational flow (1). Follow-up questions are often missing or contextually inappropriate when needed (2). Some follow-ups are attempted but are loosely related and provide little new information (3). Follow-ups are mostly relevant but lack focus or sufficient depth to elicit useful content (4). Follow-up questions are naturally connected to the response and elicit clear information, but do not reach the core issue (5). Follow-ups build logically on prior responses and elicit meaningful and detailed information (6). Follow-ups are closely aligned with the response and effectively lead the conversation to key clinical insights (7).
Similarity	The question style, transitions, and wording are far from real counseling, lacking clinical intent or strategy (1). Expressions are generally unnatural, and the counseling flow and structure differ notably from real counseling (2). Some elements resemble counseling language, but overall phrasing and structure remain awkward (3). Some expressions and transitions feel natural, but awkward sentences disrupt the flow (4). The conversation generally reflects real counseling, with minor inconsistencies in tone or flow (5). Wording and tone are mostly natural and similar to real counseling, though slight awkwardness may remain (6). Fully mirrors professional counseling style, tone, and structure with no noticeable unnaturalness (7).

a CACAD: Conversational Artificial Intelligence for Child Abuse Detection.

b This table defines the 7-point scale criteria for 3 evaluation dimensions (diversity, informativeness, and similarity) used by expert evaluators to assess CACAD–generated counseling responses. The criteria were finalized based on feedback from domain expert–generated counseling responses.

In addition to human evaluation, we also perform quantitative evaluation on 2 components of CACAD related to counseling performance, which include NQCP and abusive question detection. We use accuracy to evaluate performance on the NQCP task, and both accuracy and macro*–F*_1_-score to assess performance on abusive question detection.

#### Child Abuse Detection Evaluation

We evaluated the child abuse detection performance of the CACAD framework using the exact match (EM) [58] and macro*–F*_1_-score. EM considers a prediction correct only when all 4 child abuse category labels exactly match the ground truth, enabling assessment of consistency and agreement at the case level. The macro*–F*_1_-score computes the arithmetic mean of the *F*_1_-scores for each category to mitigate the impact of class imbalance and assess balanced performance across categories. Both metrics align with the goal of CACAD, which aims to provide comprehensive, consistent, and reliable predictive performance at the case level. In addition, we computed accuracy, precision, recall, and *F*_1_-score separately for each abuse category to provide a detailed analysis of the model. This offers a more comprehensive evaluation than metrics that only consider whether abuse is detected or not. In addition, we used the area under the risk-coverage curve [59] to evaluate selective prediction performance by quantifying the trade-off between coverage and risk under different uncertainty thresholds.

### Experimental Details

We implement the baselines using PyTorch (Meta AI) [60] and HuggingFace Transformers (Hugging Face) [61]. We conduct all experiments on 2 NVIDIA A6000 GPUs and 1 NVIDIA A100 GPU. To improve model performance, we conduct a series of experiments with various hyperparameter configurations. For PLMs, we experiment with three tasks: (1) child abuse detection, (2) abusive question detection, and (3) NQCP. For all 3 tasks, we vary the batch sizes (8, 16, 32), learning rates (1e-4, 5e-5, 1e-5, 5e-6, 1e-6), and the number of training epochs (10, 20). For all tasks except human evaluation and 5-fold cross-validation, the best checkpoint is selected based on validation performance and used to report test results. For LLMs, we use batch sizes of (2, 4), learning rates of (2e-4, 1e-5, 5e-5, 1e-6), and train for (20, 30) epochs. We set the temperature [62] to 0 and apply greedy decoding for abuse detection and for generating |follow| and |category| tokens used to control conversation flow. In the general question generation stage, we used 3 conversation examples per abuse category as in-context prompts for both CACAD and all baseline LLMs, with a temperature of 0.7. The few-shot examples were sampled from the training set. Additional experimental details for the models, including maximum sequence lengths and training curves, are provided in Multimedia Appendix 1.

### Baselines

In this study, we evaluate the performance of CACAD by comparing it with both PLMs and LLMs. For counseling, CACAD is compared against 2 instruction-tuned LLMs developed by LG AI Research and tailored for the Korean language, EXAONE-3.5‐7.8B-Instruct [63] and EXAONE-3.5-32B-Instruct-AWQ [64], and GPT-4o (version: 2024-11-20; OpenAI). As the EXAONE models exhibited the strongest performance on Korean benchmarks, they were considered the most suitable for evaluating Korean counseling scenarios [65]. For the abuse detection task, we select PLM baselines optimized for the Korean language, including KoBERT [66], KLUE-BERT-base [67], KLUE-RoBERTa-base [68], KLUE-RoBERTa-large [69], KoBigBird-BERT-base [70], KoBigBird-RoBERTa-large [71], and KoSimCSE-RoBERTa [72]. As LLM baselines, we select Qwen2.5-3B-Instruct [73], Polyglot-Ko-5.8B [74], and GPT-4o, all of which are pretrained on Korean data or capable of multilingual understanding. Among them, Qwen2.5-3B-Instruct and Polyglot-Ko-5.8B are evaluated with instruction-tuning, while GPT-4o is evaluated using a few-shot prompting approach. In addition, for the NQCP and abusive question detection tasks, we evaluate a subset of PLM baselines with strong sentence-level representation capabilities.

## Results

### Evaluation of Child Abuse Detection

Table 5 presents the experimental results comparing the performance of PLM and LLM baseline models on the child abuse detection dataset. Given the class imbalance toward “nonabuse” labels, we included a naive majority-class baseline, which achieves an EM score of 0.533. Qwen2.5-3B-Instruct achieved the highest EM score of 0.907, outperforming all PLM baselines and LLMs overall, while Polyglot-Ko-5.8B recorded a comparable EM score of 0.903. While Qwen2.5-3B-Instruct shows a slightly higher EM, according to the McNemar test [75], the difference between the two models is negligible (*P*>.99). Although PLMs achieve faster inference, LLMs attain higher accuracy, with inference latency remaining within a range suitable for real-time inference.

**Table 5.** Performance comparison of PLM^a^ and LLM^b^ baselines on child abuse detection^c^.

Model		EM^d^	macro*–F*_1_-score	Latency (s)
Naive majority-class baseline		0.533	0.000	—^e^
PLM				
KoBERT		0.872	0.893	*0.014*
KLUE-BERT-base		0.891	0.932	0.015
KLUE-RoBERTa-base		0.875	0.918	0.015
KLUE-RoBERTa-large		0.885	0.928	0.032
KoBigBird-BERT-base		0.888	0.931	0.016
KoBigBird-RoBERTa-large		0.882	0.919	0.034
KoSimCSE-RoBERTa		0.875	0.912	0.015
LLM				
GPT-4o		0.891	0.926	1.82
Polyglot-Ko-5.8B		0.903	*0.942*	2.84
Qwen2.5-3B-Instruct		*0.907*	0.939	3.94

a PLM: pretrained language model.

b LLM: large language model.

c Latency is reported in seconds, and the best results are italicized.

d EM: exact match.

e Not applicable.

### Evaluation of Child Abuse Counseling

Table 6 shows the results of the NQCP prediction across the 4 abuse types. KLUE-RoBERTa-large achieved the highest accuracy in the neglect and physical categories, recording 0.944 and 0.866, respectively. KLUE-BERT-base showed the best performance in the emotional abuse category with an accuracy of 0.915. For sexual abuse, both KLUE-BERT-base and KoSimCSE-RoBERTa attained the top accuracy of 0.903. These results demonstrate that NQCP prediction enables CACAD to generate appropriate questions during counseling and to terminate the conversation at contextually appropriate points. For the abusive question detection task, Table 7 shows that KoSimCSE-RoBERTa outperformed all other models, achieving the highest accuracy and macro*–F*_1_-score of 0.980. Furthermore, an additional evaluation was conducted on 50 out-of-distribution questions composed of realistic adversarial prompts and near-miss cases generated by 2 human experts, and the model demonstrated perfect detection performance across all cases. This result highlights its effectiveness in identifying inappropriate or unethical questions during counseling, and no fallback mechanisms were triggered during the entire human expert counseling evaluation. Table 8 presents the average human evaluation scores from 3 domain experts, following the 1‐7 point scale scoring guidelines. Our proposed framework CACAD, which integrates the EXAONE-3.5-32B-Instruct-AWQ model with next question category recommendations, recorded the highest scores across all criteria: diversity 5.00, informativeness 5.67, and similarity 5.67. In contrast, models using only prompt tuning without question recommendation showed generally lower performance. In particular, EXAONE-3.5‐7.8B-Instruct and EXAONE-3.5-32B-Instruct-AWQ recorded lower diversity scores (3.00 and 4.00) and informativeness scores (2.33 and 3.33) compared to CACAD. These results indicate that its follow-up questions failed to elicit meaningful information or struggled to ask sufficiently diverse questions for identifying abuse indicators. Both models showed substantial improvement when the next question recommendation strategy was applied. This gain appears to reflect the structural characteristics of real counseling conversations. In actual sessions, question sequences tend to follow consistent patterns depending on the child’s responses, and counselors often repeat semantically similar questions across cases. Such regularities likely enabled the category-based approach to more effectively capture the natural flow of real counseling interactions. While the NQCP and abusive question detection modules exhibit negligible inference latency, CACAD incurs additional latency due to the verification of category and follow-up decisions, as well as the question generation process (≈5.9‐9.0 s per conversational turn). As a result, CACAD shows higher latency than the baselines but achieves superior performance. In postevaluation interviews, evaluators reported no perception of latency-based unblinding. Table 9 shows the interrater reliability among the 3 human evaluators measured using ICC (3,k). Following established guidelines in the reliability analysis literature, ICC values between 0.75 and 0.90 are interpreted as good reliability, while values above 0.90 indicate excellent reliability [76]. The results show excellent reliability for diversity (0.9091) and informativeness (0.9181), and good reliability for similarity (0.7952). These findings indicate a high level of consistency among evaluators, supporting the reliability of the human evaluation results.

**Table 6.** Accuracy comparison of NQCP^a^ models across 4 abuse types (neglect, emotional, physical, and sexual)^b^.

Category and model		Accuracy	Latency (s)
Neglect			
KLUE-BERT-base		0.939	*0.007*
KLUE-RoBERTa-large		*0.944*	0.013
KoSimCSE-RoBERTa		0.937	*0.007*
KoBigBird-RoBERTa-large		0.942	0.025
Emotional			
KLUE-BERT-base		*0.915*	0.007
KLUE-RoBERTa-large		0.913	0.013
KoSimCSE-RoBERTa		0.908	*0.006*
KoBigBird-RoBERTa-large		0.914	0.017
Physical			
KLUE-BERT-base		0.852	*0.007*
KLUE-RoBERTa-large		*0.866*	0.013
KoSimCSE-RoBERTa		0.850	*0.007*
KoBigBird-RoBERTa-large		0.846	0.017
Sexual			
KLUE-BERT-base		*0.903*	*0.006*
KLUE-RoBERTa-large		0.896	0.014
KoSimCSE-RoBERTa		*0.903*	0.007
KoBigBird-RoBERTa-large		0.900	0.016

a NQCP: next question category prediction.

b Latency is reported in seconds, and the best results are italicized.

**Table 7.** Accuracy and macro*–F*_1_-score comparison of abusive question detection models^a^.

Model	Accuracy	macro*–F*_1_-score	Latency (s)
KLUE-BERT-base	0.970	0.970	0.011
KLUE-RoBERTa-large	0.970	0.970	0.013
KoSimCSE-RoBERTa	*0.980*	*0.980*	*0.007*

a Latency is reported in seconds, and the best results are italicized.

**Table 8.** Averaged human evaluation scores for counseling performance^a^^,^^b^.

Model	Diversity	Informativeness	Similarity	Latency (s)
EXAONE-3.5‐7.8B-Instruct	3.00	2.33	4.00	*3.2-5.0*
EXAONE-3.5-32B-Instruct-AWQ	4.00	3.33	3.67	3.3-5.3
CACAD^b^ (7.8B)	5.00	4.67	4.33	5.9-8.2
CACAD (32B)	*5.00*	*5.67*	*5.67*	5.9-9.0

a CACAD denotes a baseline model augmented with the next question category prediction and the abusive question detection module, with different model sizes (7.8B and 32B). Response latency is reported in seconds, and the best results are italicized.

b CACAD: Conversational Artificial Intelligence for Child Abuse Detection.

**Table 9.** Results of the interrater reliability analysis among 3 domain experts^a^.

Criterion	Rater 1	Rater 2	Rater 3	ICC^b^ (3, k)
Diversity	4, 4, 5, 5	3, 4, 5, 5	2, 4, 5, 5	0.9091
Informativeness	1, 3, 5, 6	3, 4, 5, 6	3, 3, 4, 5	0.9181
Similarity	5, 5, 4, 6	4, 3, 5, 6	3, 3, 4, 5	0.7952

a Rows represent counseling quality evaluation criteria (diversity, informativeness, and similarity), while columns rater 1‐3 indicate the 7-point scale score distributions assigned by each evaluator. For each rater, scores are presented in the order of EXAONE-3.5‐7.8B-Instruct, EXAONE-3.5-32B-Instruct-AWQ, CACAD (7.8B), and CACAD (32B).

b ICC: intraclass correlation coefficient.

### Evaluation of Child Abuse Detection by Category

Table 10 presents a comparison of model performance for each of the 4 abuse types, based on accuracy, precision, recall, and *F*_1_-score. For neglect detection, Polyglot-Ko-5.8B achieved the highest performance with an *F*_1_-score of 0.907. In the case of emotional abuse, Qwen2.5-3B-Instruct outperformed other baselines, recording an accuracy of 0.950 and an *F*_1_-score of 0.915. For physical abuse, GPT-4o demonstrated the best performance with an *F*_1_-score of 0.978. In the case of sexual abuse, all 3 models achieved 1.0 across all evaluation metrics. The values in parentheses represent the results of a 5-fold cross-validation conducted under the same experimental setting to verify that the observed performance is not attributable to overfitting. Although a slight decrease in performance is observed for sexual abuse detection under cross-validation, all models still maintain high detection accuracy, confirming the robustness and consistency of the original results. However, these results should be interpreted with caution, as the relatively small sample size of sexual abuse (n=33). Moreover, the overall high performance in detecting physical and sexual abuse can be explained by the nature of conversations in such cases, where counselors ask direct questions about concrete situations and children tend to give brief, binary responses, often in the form of “yes” or “no.” This interaction pattern provides clear cues for the model to make accurate classifications. In contrast, neglect and emotional abuse are more challenging to detect, as children’s expressions in these cases tend to be indirect and heavily reliant on context. Nevertheless, Qwen2.5-3B-Instruct and Polyglot-Ko-5.8B, which were instruction-tuned specifically for abuse detection, outperformed GPT-4o in these categories. In particular, as emotional abuse accounts for 43.1% of all child abuse cases in Korea [14], this highlights the practical value of CACAD’s classification model in enhancing detection performance in this critical area.

**Table 10.** Per-category performance of each model on child abuse detection (Acc.: Accuracy; Prec.: Precision; Rec.: Recall; *F*_1_: *F*_1_-score)^a^.

Category and metric		GPT-4o	Polyglot-Ko-5.8B	Qwen2.5-3B-Instruct
Neglect				
Acc.		0.953 (0.959)	*0.975 (0.974)*	0.969 (0.970)
Prec.		0.810 (*0.935)*	0.867 (0.920)	*0.878* (0.923)
Rec.		0.829 (0.796)	*0.951 (0.914)*	0.878 (0.880)
*F*_1_		0.819 (0.859)	*0.907 (0.917)*	0.878 (0.901)
Emotional				
Acc.		0.941 (0.937)	0.938 *(0.953)*	*0.950* (0.950)
Prec.		0.874 (0.845)	0.896 *(0.913)*	*0.935* (0.894)
Rec.		*0.938 (0.952)*	0.896 (0.921)	0.896 (0.934)
*F*_1_		0.905 (0.895)	0.896 *(0.917)*	*0.915* (0.914)
Physical				
Acc.		*0.991 (0.986)*	0.984 (0.984)	0.984 (0.985)
Prec.		*0.971* (0.943)	0.932 (0.948)	0.944 *(0.953)*
Rec.		0.985 *(0.988)*	*1.000 (0.969)*	0.985 (0.973)
*F*_1_		*0.978 (0.966)*	0.964 (0.959)	0.964 (0.963)
Sexual				
Acc.		1.000 *(0.998)*	1.000 (0.997)	1.000 (0.997)
Prec.		1.000 *(0.990)*	1.000 (0.976)	1.000 (0.986)
Rec.		1.000 *(0.990)*	1.000 (0.986)	1.000 (0.983)
*F*_1_		1.000 *(0.990)*	1.000 (0.981)	1.000 (0.984)

a Results from 5-fold cross-validation are reported in parentheses. The best results are italicized.

### Evaluation of Classification Performance Under Uncertainty Thresholds

**Figure 3.**
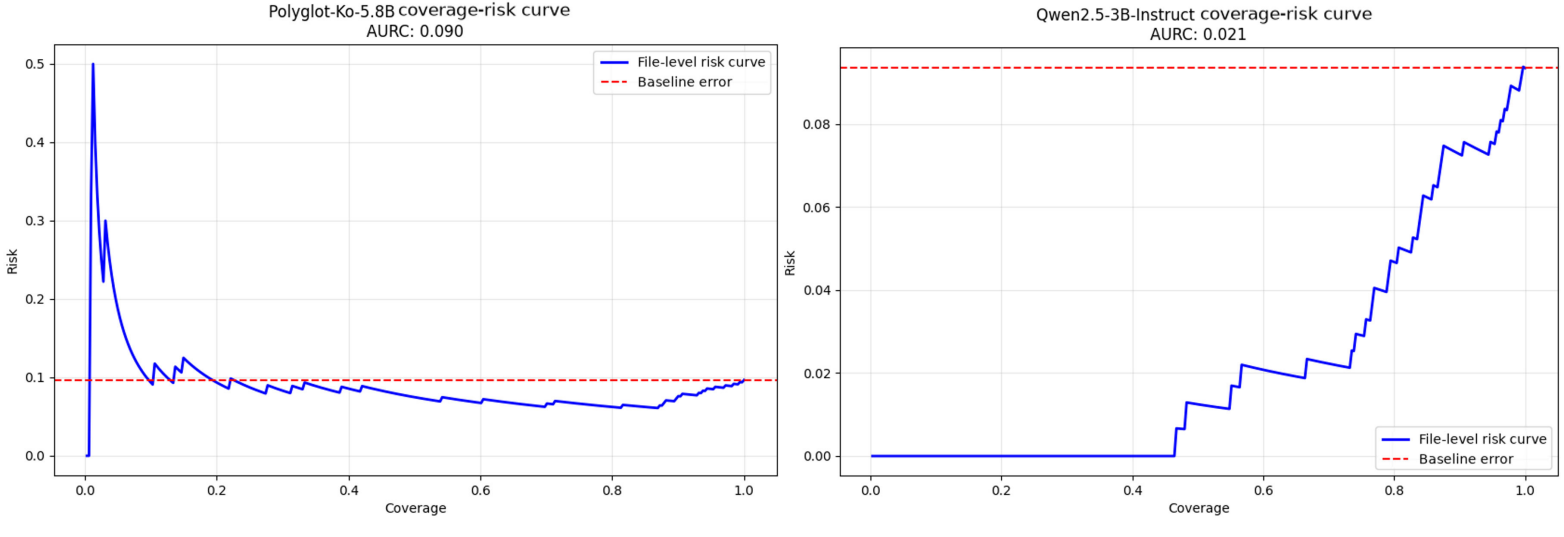
Coverage-risk curves on child abuse detection. The curves show the trade-off between coverage (proportion of automatically processed cases) and risk (cumulative error rate over the nondeferred cases). The dashed red line indicates the baseline error when all cases are handled automatically. AURC: area under the risk-coverage curve.

Table 11 presents the selective prediction performance of Qwen2.5-3B-Instruct and Polyglot-Ko-5.8B, evaluated across multiple uncertainty thresholds based on MSP and entropy, illustrating the trade-off between system performance and coverage as the threshold varies. In the CACAD scenario, we assume that the model refers a case to a human counselor as “pending review” when the predicted probability falls below the threshold in MSP or when the uncertainty value exceeds the threshold in entropy. We evaluate the system by comparing EM across 3 counselor accuracy levels (100%, 90%, and 80%). This analysis provides a performance range under more realistic deployment scenarios where human error may occur. Note that macro*–F*_1_-score is calculated only for the 100% accuracy scenario, as the specific distribution of incorrect human predictions cannot be determined for the 90% and 80% cases. Adjusting the thresholds increases the pending review ratio and thereby improves performance in both methods. However, Qwen2.5-3B-Instruct achieves clear performance gains during this process; under the entropy-based approach, when 53.58% of the data are allocated to a human counselor, its classification accuracy reaches 100%. In contrast, Polyglot-Ko-5.8B limits the pending review ratio to only 20.56%, resulting in limited performance improvement. This difference arises from the distribution of uncertainty across the 2 models. Qwen2.5-3B-Instruct outputs a relatively diverse range of uncertainty values, which makes threshold adjustment effective, whereas Polyglot-Ko-5.8B concentrates its predictions at extreme confidence levels. In particular, Polyglot-Ko-5.8B frequently outputs a softmax probability of exactly 1 under MSP and an entropy value of exactly 0, and this excessive confidence prevents selective prediction strategies based on threshold adjustment from functioning effectively. To quantitatively assess the reliability of the probability estimates used for deferral decisions, we additionally report expected calibration error (ECE) [77] and Brier score [78]. Table 12 reports the calibration performance of each model across the 4 child abuse categories. Overall, Qwen2.5-3B-Instruct shows lower ECE and Brier scores than Polyglot-Ko-5.8B across most abuse categories, suggesting that the confidence levels produced by the model are more consistent with its actual prediction accuracy. In particular, for the physical and sexual abuse categories, both calibration metrics attain notably low values, suggesting that the model produces relatively stable and well-calibrated probability estimates for these categories. Figure 3 shows the effect of selective prediction strategies in the child abuse detection task using coverage-risk curves. In this figure, coverage denotes the proportion of cases that are automatically processed by the model without being deferred to a human counselor based on an uncertainty criterion, while risk represents the cumulative error rate computed over these automatically processed cases. Each point on the coverage-risk curve reflects changes in the automatically handled case set and the corresponding error risk as the uncertainty threshold varies. Qwen2.5-3B-Instruct achieved a lower area under the risk-coverage curve (0.021) than Polyglot-Ko-5.8B (0.090), indicating more effective uncertainty-based deferral. As coverage decreases, Qwen2.5-3B-Instruct exhibits a substantial reduction in risk, whereas Polyglot-Ko-5.8B shows a limited decrease in risk due to its prediction confidence being concentrated at extreme values, which hinders gradual case deferral through threshold adjustment.

**Table 11.** Threshold-based child abuse detection performance of uncertainty-aware triage methods^a^.

Method and threshold		Pending review	Pending review ratio (%)	H100% EM^b^	H100% macro*–F*_1_-score	H90% EM	H80% EM
Polyglot MSP^c^							
0.5975		0	0.00	0.903	0.942	0.903	0.903
0.8590		5	1.56	0.910	0.949	0.908	0.907
0.9755		10	3.12	0.916	0.952	0.913	0.910
0.9960		20	6.23	0.922	0.954	0.916	0.910
1.0000		42	13.08	0.947	0.968	0.934	0.921
Polyglot entropy							
0.6745		0	0.00	0.903	0.942	0.903	0.903
0.4075		5	1.56	0.910	0.949	0.908	0.907
0.1155		10	3.12	0.916	0.952	0.913	0.910
0.0265		20	6.23	0.922	0.954	0.916	0.910
0.0010		38	11.84	0.941	0.965	0.929	0.917
0.0000		66	20.56	0.972	0.985	0.951	0.931
Qwen MSP							
0.0000		0	0.00	0.907	0.939	0.907	0.907
0.8600		20	6.23	0.932	0.957	0.925	0.919
0.9465		40	12.46	0.935	0.958	0.922	0.910
0.9720		60	18.69	0.954	0.968	0.941	0.922
0.9805		81	25.23	0.963	0.974	0.953	0.928
0.9880		100	31.15	0.972	0.982	0.953	0.922
0.9925		120	37.38	0.988	0.988	0.950	0.913
0.9950		142	44.24	0.991	0.996	0.946	0.902
0.9965		165	51.40	0.994	0.997	0.942	0.891
0.9980		181	56.39	1.000	1.000	0.944	0.887
1.0000		321	100.00	1.000	1.000	0.900	0.800
Qwen entropy							
0.6925		0	0.00	0.907	0.939	0.907	0.907
0.4575		18	5.61	0.932	0.957	0.926	0.920
0.2085		42	13.08	0.938	0.960	0.928	0.915
0.1210		60	18.69	0.953	0.968	0.941	0.922
0.0980		80	24.92	0.963	0.974	0.950	0.925
0.0660		100	31.15	0.972	0.982	0.953	0.922
0.0440		120	37.38	0.978	0.988	0.950	0.913
0.0340		140	43.61	0.991	0.996	0.947	0.903
0.0265		160	49.84	0.994	0.997	0.944	0.894
0.0205		172	53.58	1.000	1.000	0.946	0.893
0.0000		321	100.00	1.000	1.000	0.900	0.800

a We report the pending review ratio and performance across varying uncertainty thresholds for each method. Qwen refers to Qwen2.5-3B-Instruct, and Polyglot refers to Polyglot-Ko-5.8B. H100%, H90%, and H80% EM assume 100%, 90%, and 80% human accuracy on deferred cases.

b EM: exact match.

c MSP: maximum softmax probability.

**Table 12.** Model calibration performance measured by expected calibration error and Brier score across child abuse categories.

Model and category		ECE^a^	Brier score
Polyglot-Ko-5.8B			
Neglect		0.0235	0.0231
Emotional		0.0614	0.0613
Physical		0.0160	0.0140
Sexual		6.23 × 10^–7^	1.25 × 10^–10^
Qwen2.5-3B-Instruct			
Neglect		0.0235	0.0251
Emotional		0.0392	0.0458
Physical		0.0133	0.0152
Sexual		0.0011	3.49 × 10^–5^

a ECE: expected calibration error.

## Discussion

### Principal Results and Interpretation

**Figure 4.**
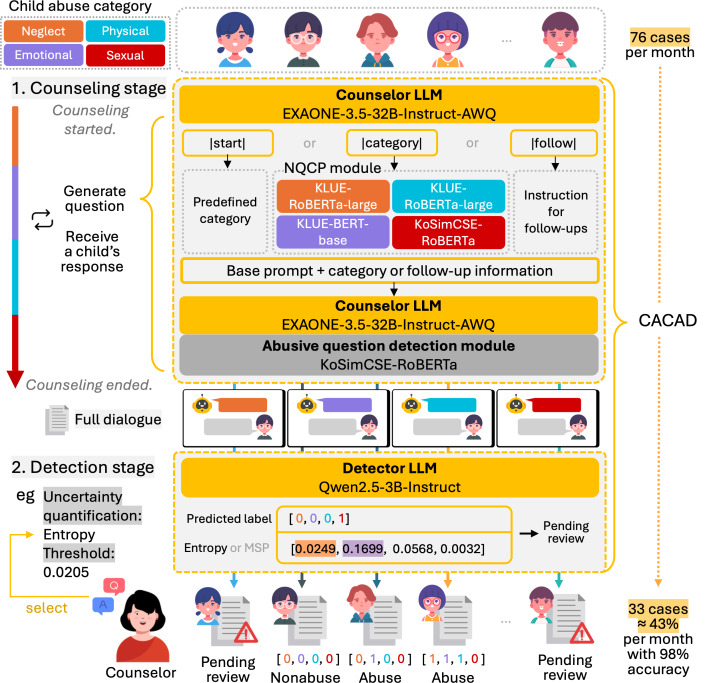
Overall workflow of the Conversational Artificial Intelligence for Child Abuse Detection framework, illustrating the entire process with the final model selected through empirical evaluation. CACAD: Conversational Artificial Intelligence for Child Abuse Detection; LLM: large language model; NQCP: next question category prediction.

The experimental results of this study demonstrate that CACAD can serve as a practical tool for supporting counselors in the context of child abuse detection. As illustrated in Figure 4, CACAD consists of a 2-stage framework comprising a counseling stage and a detection stage, with multiple safety mechanisms integrated throughout the workflow. In the child abuse detection task, CACAD achieved an EM of 0.907 and a macro*–F*_1_-score of 0.939, exhibiting competitive performance compared to baseline models based on PLMs and LLMs. Although Qwen2.5-3B-Instruct and Polyglot-Ko-5.8B showed comparable performance in terms of EM, from the perspective of uncertainty quantification, Polyglot-Ko-5.8B tends to produce overconfident predictions and exhibits less stable risk reduction as coverage decreases. In contrast, Qwen2.5-3B-Instruct shows a broader distribution of uncertainty, enabling more reliable threshold-based decision and selective prediction, and was therefore selected as the final model. Notably, the superior performance of instruction-tuned models in categories such as neglect and emotional abuse, where children’s disclosures tend to be indirect and highly context-dependent, highlights the potential applicability of CACAD in real counseling settings. The results of the human evaluation on counseling stages further support the effectiveness of CACAD. CACAD outperformed all baseline models in terms of question diversity (5.00), informativeness (5.67), and similarity (5.67) to real counseling interactions. These human evaluation results indicate that CACAD can explore diverse topics and effectively elicit meaningful information while maintaining the overall flow of counseling. However, some redundancy in expression and slight awkwardness remain, which may prevent children from perceiving the interaction as fully natural and could affect initial trust. Therefore, CACAD should be understood not as a replacement for counselors, but as a supportive tool that, under professional supervision, can enhance counselor trust and provide children with a safer and more consistent counseling experience. The design of CACAD aligns with forensic interviewing principles that prioritize open-ended questions over accusatory or hypothesis-driven inquiries. The use of the |follow| token for follow-up questioning, category-based transitions via the |category| token, and learned conversation termination through the |end| token collectively prevents the model from excessively pursuing questions that reinforce a specific abuse hypothesis. Furthermore, the abusive question detection module proactively identifies and blocks suggestive, leading, or coercive questions in real time, thereby removing questions that could contaminate children’s disclosures. This design is consistent with established forensic interviewing guidelines, such as the National Institute of Child Health and Human Development protocol, which emphasizes collecting information primarily through children’s spontaneous narratives [79]. Overall, the reported performance metrics indicate that both children and counselors can rely on CACAD not as an autonomous decision-making agent, but as a decision support tool. In particular, the explicit uncertainty quantification and design of the system to request expert intervention when predefined thresholds are exceeded enable responsible and accountable deployment in real counseling environments.

### Deployment Considerations

In real-world settings, CACAD should be deployed not as an autonomous system, but as a decision support tool operating under expert supervision. System access should be clearly separated between counselors and children through role-based access control, and counselors should be able to monitor all sessions in real time. At the conclusion of each counseling session, CACAD should provide both its inferred abuse risk assessment and associated uncertainty estimates, and cases identified as high risk or requiring further review should be promptly reported to the counselor. From an operational perspective, all counseling interactions and model inference outputs should be recorded as audit logs to enable subsequent review and accountability. In addition, incident response and escalation protocols should be clearly defined so that timely expert intervention can be initiated when potential risk signals are detected. Data protection is also a critical consideration. CACAD should process only the minimum amount of conversational information necessary to support counseling and abuse detection, and it should not use personally identifiable information. Data access must be strictly governed under the principle of purpose limitation, and all data should not be used for purposes beyond those explicitly defined. These operational principles are essential for ensuring the trustworthiness and social acceptability of systems that handle sensitive counseling data.

### Limitations

CACAD presents a new direction for child abuse detection by directly conducting counseling and making judgments, but several limitations remain. First, CACAD operates exclusively on text, which presents a significant clinical limitation by failing to account for nonverbal and multimodal cues, such as physical demeanor, pauses, crying, and vocal tone, that are vital to pediatric counseling. Second, the human evaluation was conducted using expert role-playing, which may not fully capture the complex response patterns frequently observed in real children, such as resistance, verbal regression, or fragmented and noncontinuous utterances. While this form of proxy evaluation is useful for validating an early-stage prototype, it does not fully represent real clinical counseling environments. Additionally, conducting only 5 sessions per system in the evaluation resulted in a small sample size, which limits the statistical weight and generalizability of the evaluation results. Third, CACAD has system-level limitations, including constraints in capturing conversational context and real-time response latency, due to design choices that prioritize safety and operational stability. The regeneration and fallback mechanisms introduced to ensure real-time safety inherently involve design trade-offs. The use of predefined questions may fail to fully reflect the current conversational context, and transitions to the next question category may restrict further information elicitation in situations where additional exploration is warranted. Furthermore, the 5.9‐ to 9.0-second latency per conversational turn resulting from this model design may reduce engagement or increase session abandonment among children and adolescents. Fourth, the use of a perfectly balanced abusive question detection dataset may fail to reflect real-world clinical deployment, where nonabusive queries are more prevalent. Consequently, the experimental design potentially obscures the actual false positive rate, which may result in frequent fallback activations and degrade generative flexibility. Moreover, due to ethical constraints, we did not conduct experiments that intentionally prompt the LLM to generate abusive or harmful questions. As a result, the quantitative evaluation of potentially risky model outputs is limited. Although no explicit hallucinations were observed during the role-play evaluations, the possibility of such issues arising in real-world settings cannot be completely ruled out. Fifth, this study was conducted using a single dataset, and external validation to fully assess the model’s generalizability remains limited. Specifically, the limited positive sample size in the sexual abuse category (n=33), along with the dataset-specific characteristics, such as clearly structured questions and “yes or no” response patterns from children, may limit the generalizability of the results. While we attempted to mitigate overfitting through category-level performance analysis and cross-validation, additional evaluation on independent counseling datasets was not performed and remains an important direction for future work. Furthermore, since the LLMs used in this study were pretrained on large-scale public corpora, the possibility of data contamination cannot be entirely dismissed. Although our task uses annotated labels not included in the original dataset, and zero-shot evaluations without task-specific fine-tuning showed relatively low performance (Polyglot-Ko-5.8B: EM 0.5327, macro*–F*_1_-score 0.000; Qwen2.5-3B-Instruct: EM 0.645, macro*–F*_1_-score 0.632), partial overlap may still have contributed to the reported performance. Finally, this study is based on Korean-language child and adolescent counseling data, and experimental validation in other languages or national counseling contexts was not included. Consequently, the direct generalization of our findings to other linguistic or cultural settings is limited, and further experiments using non-Korean counseling datasets are required. In addition, operational factors present in real counseling environments, such as referral costs and institution-specific human resource constraints, were not quantitatively modeled, which constitutes another limitation of this study. Furthermore, this study does not account for developmental variations between children and adolescents, which may influence how the framework interprets specific linguistic or behavioral cues.

### Future Work

Future research should focus on deploying CACAD in real counseling environments to obtain long-term and empirical evidence of its effectiveness. To enhance clinical utility, the system should be improved to dynamically adjust the sequence of abuse-type exploration based on the child’s responses, and early termination strategies should also be implemented to minimize unnecessary questioning. In addition, optimizing the model and system architecture to reduce latency is essential for ensuring natural and seamless responsiveness in real-time interaction. Critically, when a child exhibits immediate high-risk signals, such as self-harm or suicidal ideation during the counseling process, waiting for the conversation to conclude naturally may pose serious risks. Therefore, it is necessary to design a rule-based keyword interruption protocol that can promptly detect and request expert intervention. In addition, considering the potential loss of generative flexibility from excessive fallback activation, future work should consider naturalistically imbalanced abusive question detection data distributions to ensure accurate estimation of false-positive rates. Moreover, counseling support systems that leverage commercial or open-source LLMs, including CACAD, may continue to raise concerns regarding the protection of sensitive information disclosed by children. To address these issues, future work should further investigate privacy-centered system design and operational strategies, such as strengthened data deidentification, strict access control and usage logging, and the establishment of local inference environments. Such efforts will be essential for enabling CACAD to function as a trustworthy counseling support tool in real-world settings.

### Conclusions

In this study, we propose CACAD, a conversational AI framework that leverages an LLM to conduct child counseling and detect potential child abuse. CACAD is designed to generate contextually appropriate questions for 4 abuse types by incorporating prior conversational context and the predicted category of the next counselor question. In addition, we experimentally demonstrate that instruction-based learning enables effective abuse-type detection. To ensure safety during counseling, CACAD integrates an abusive-question detection module and incorporates uncertainty quantification techniques in the abuse detection stage. This design allows the system to proactively filter inappropriate or unethical questions during interaction, while quantitatively assessing uncertainty in detection outcomes to identify cases that require expert review. Taken together, these results suggest that LLM-based conversational systems can be applied to sensitive domains such as child abuse detection through structured designs that incorporate multistage safety mechanisms and human-in-the-loop oversight.

## Supplementary material

10.2196/86536 Multimedia Appendix 1 Maximum sequence length and training time for finalized models on each module, and abuse detection large language model training loss curve.

## Abbreviations

AI: artificial intelligence
CACAD: Conversational Artificial Intelligence for Child Abuse Detection
ECE: expected calibration error
EM: exact match
ICC: intraclass correlation coefficient
LLM: large language model
MLC: multilabel classification
MSP: maximum softmax probability
NQCP: next question category prediction
PLM: pretrained language model
