# Supplementary material for: Conversational AI for Child Abuse Detection Through Multistage Counseling: Model Development and Validation Study
Source: J Med Internet Res. 2026 Jul 10;28:e86536. doi: 10.2196/86536 (PMC13354129; doi:10.2196/86536)

Max sequence length and training time for finalized models on each module:

| Module | Model | Max sequence | Training time  per 1 epoch (hour) |
| --- | --- | --- | --- |
| NQCP | KLUE-BERT-base | 512 | 0.018 |
|  | KLUE-RoBERTa-large | 512 | 0.068 |
|  | KoSimCSE-RoBERTa | 512 | 0.020 |
| Abusive question detection | KoSimCSE-RoBERTa | 512 | 0.004 |
| Child abuse detection | Polyglot-Ko-5.8B | 2048 | 0.592 |
|  | Qwen2.5-3B-Instruct | 2048 | 0.322 |

Polyglot-Ko-5.8B training curve:


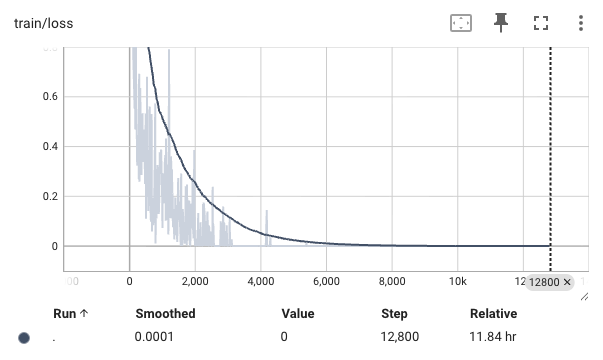


### Qwen2.5-3B-Instruct training curve:


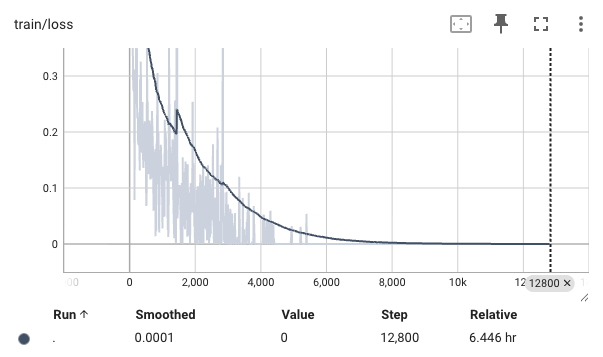

Supplement: Multimedia Appendix 1 [file jmir-v28-e86536-s001.docx]
